# Determination of ^226^Ra, ^232^Th, ^40^K, ^235^U and ^238^U activity concentration and public dose assessment in soil samples from bauxite core deposits in Western Cameroon

**DOI:** 10.1186/s40064-016-2895-9

**Published:** 2016-08-04

**Authors:** Eric Jilbert Mekongtso Nguelem, Maurice Moyo Ndontchueng, Ousmanou Motapon

**Affiliations:** 1 Department of Physics, The University of Douala, P.O. Box 24157, Douala, Cameroon; 2 The National Radiation Protection Agency, P.O. Box 33732, Yaoundé, Cameroon

## Abstract

Determination of activity concentrations in twenty five (25) soil samples collected from various points in bauxite ore deposit in Menoua Division in Western of Cameroon was done using gamma spectrometry based Broad Energy Germanium (BEGe6530) detector. The average terrestrial radionuclides of ^40^K, ^226^Ra, ^232^Th, ^235^U and ^238^U were measured as 671 ± 272, 125 ± 58, 157 ± 67, 6 ± 3 and 99 ± 69 Bq kg^−1^, respectively. The observed activity concentrations of radionuclides were compared with other published values in the world. The outdoor absorbed dose rate in air varied from 96.1 to 321.2 nGy h^−1^ with an average of 188.2 ± 59.4 nGy h^−1^. The external annual effective dose rate and external hazard index were estimated as 0.23 ± 0.07 mSv year^−1^ for outdoor, 0.92 ± 0.29 mSv year^−1^ for indoor and 1.13 for the external hazard index, respectively. These radiological safe parameters were relatively higher than the recommended safe limits of UNSCEAR. Consequently, using of soil as building material might lead to an increase the external exposure to natural radioactivity and future applications research need to be conducted to have a global view of radioactivity level in the area before any undergoing bauxite ore exploitation.

## Background

Natural radioactive materials under certain conditions in the environmental matrix can reach hazardous radiological levels (Selvasekarapandian et al. 1999). Geological and hydrogeological conditions can sometimes lead to their enrichment in the environment, creating mineral deposits over a geological time scale. It is formed due to weathering of rocks in the earth’s crust and deposition of eroded materials into depressions (Tufail et al. 2013). Exploration of these natural mineral (case of uranium, bauxite etc.…) raises concerns related to waste management and environmental contamination by NORM (IAEA 1996). Site remediation after bauxite mining and milling also proves to be a major issue of radiological protection with a risk essentially associated with the daughter products of uranium and thorium (Saïdou et al. 2011).

The western region of Cameroon is known to have large bauxite ore deposits. Since 1950, previous investigation was carried out by foreign office of geological mining research. The objective of this previous investigation was to evaluate the mineral potential of the western region of Cameroon. The results of their investigations have shown that there are about 54,000,000 tons of Bauxite ore deposit found in Menoua division of the western region.

The project of exploiting the site is likely to become a reality soon. Investigation of possible radiological impact before mining exploration has not been undertaken yet. Population living in that area uses sundry bricks made of soil for house constructions. Many studies worldwide also reveal the impact of natural mineral mining and milling in the environment has been a great of interest (Winkelmann et al. 2001; Carvalho et al. 2007; Saïdou et al. 2011; Ndontchueng et al. 2014). However, this impact cannot be well established without performing radioactivity measurements onsite and in the vicinity of the site prior to mining activities. Natural radioactivity measurements in soil using gamma spectrometry allow the implementation of precautionary measures whenever the dose is found to be above the recommended limits. The present work aimed to (1) measure activity concentrations of ^226^Ra, ^232^Th, ^40^K, ^238^U and ^235^U in top surface soil samples collected in bauxite ores deposit site in Menoua Division of western Cameroon, (2) estimate the dose rates caused by external exposure from terrestrial radionuclides in soil samples and (3) evaluate radiological hazardous of soil samples.

## Methods

### Geology of the area and type of bauxite ore deposit

The study site was Fongo-Tongo, located on the highlands of Western Cameroon specifically the South-western side of Bambouto-Mountains. The area is characterized by low altitudes (1300–1600 m) and medium altitudes (1600–2000 m). The vegetation is strongly influenced by anthropic activities and the crops cultivated include maize, beans, potatoes and tea (Wouatong et al. 2014). The soils are *ferrallatic andic* and *ferrallitic* battleships according to CPCS (Centre Pedologique de Classification des Sols) classification. The petrography of this location consists of trachytes, welded ignimbrite, non-welded ignimbrite, basalts and granites. Rocks types in the area are essentially the basement rocks (orthogneiss, gneiss, migmatites and amphibolites) and various volcanic rocks (basalts, trachytes, phonolites, tuffs, breccia and ignimbrites). The mean chemical composition of these basalts is as follow: 15.9 % of Al_2_O_3_, 13.5 % of Fe_2_O_3_ and 44.6 % of SiO_2_. The bauxite ores deposits in this area are developed exclusively from the Miocene aphyric or porphyric basalts.

### Sample collection and preparation for radiometric analysis

Field reconnaissance survey was based on the topography. This enabled us to identify the bauxite ore deposits site in the region. To cover the study area and to observe a significant local spatial variation in terrestrial radioactivity, each sampling location was considered as being overlaid by a 25 × 25 m grid, subdivided in 15 cells of 3 × 5 m and a minimum distance between one another was 1000 m. Within each grid, different samples were randomly collected and mixed to obtained composite samples. Each of these grids was marked using a global positioning system (GPS) as shown in Table 1. Total of twenty five (25) composite soil samples were randomly collected at a typical depth of about 10–15 cm from the top surface layer. At the laboratory, the samples were dehumidified for about 24 h at ambient temperature to provide a stable homogeneous mixture and then dried in an oven at 105 °C until the complete removal of the moisture of the samples. Then, the dried samples were grinded, sieved through a 2 mm wire mesh to obtain a uniform particles size and then placed in 120 ml of plastic container and weighted. The weighed containers were hermetically sealed and stored for 30 days to capture ^222^Rn gas and ensure secular equilibrium between ^238^U and ^226^Ra, and between ^232^Th and ^228^Th has been achieved.

**Table 1:** Activity concentration of ^40^K, ^226^Ra, ^232^Th, ^235^U and ^238^U measured in soil samples together with radiological health

Sample code	Activity concentration (Bq kg^−1^)					Radiological health parameters				
	^40^K	^226^Ra	^232^Th	^235^U	^238^U	Ra_eq_ (Bq kg^−1^)	AD (nGy h^−1^)	OAED (mSv year^−1^)	IAED (mSv year^−1^)	H_ex_
SO1	1269 ± 127	86 ± 4	95 ± 4	4 ± 2	94 ± 33	320.9	150.7	0.18	0.74	0.87
SO2	998 ± 29	94 ± 2	130 ± 3	6 ± 1	122 ± 24	357.8	164.0	0.20	0.80	0.97
SO3	1004 ± 41	88 ± 4	88 ± 2	4 ± 1	85 ± 30	292.7	136.4	0.17	0.67	0.79
SO4	1200 ± 98	119 ± 5	1664 ± 7	5 ± 1	115 ± 28	450.1	205.9	0.25	1.01	1.22
SO5	135 ± 2	154 ± 2	144 ± 4	7 ± 0	150 ± 9	371.1	164.1	0.20	0.80	1.00
SO6	240 ± 4	97 ± 4	78 ± 6	6 ± 1	122 ± 24	228.5	102.6	0.13	0.50	0.62
SO7	615 ± 83	116 ± 4	59 ± 6	5 ± 0	98 ± 7	247.6	114.9	0.14	0.56	0.67
SO8	316 ± 12	62 ± 2	117 ± 2	3 ± 1	70 ± 20	253.9	112.6	0.14	0.55	0.69
SO9	652 ± 25	199 ± 14	181 ± 7	ND	ND	508.6	228.7	0.28	1.12	1.37
SO10	609 ± 22	175 ± 12	217 ± 4	ND	160 ± 20	533.9	238.1	0.29	1.17	1.44
SO11	868 ± 45	118 ± 3	231 ± 5	ND	98 ± 30	515.4	230.3	0.28	1.13	1.39
SO12	799 ± 41	288 ± 21	256 ± 10	ND	215 ± 40	715.9	321.2	0.39	1.58	1.93
SO13	831 ± 39	106 ± 5	192 ± 5	ND	102 ± 25	445.4	199.9	0.25	0.98	1.20
SO14	678 ± 21	172 ± 8	200 ± 9	ND	163 ± 17	510. 5	228.7	0.28	1.12	1.38
SO15	800 ± 41	108 ± 5	206 ± 9	ND	97 ± 29	464.7	207.9	0.25	1.02	1.25
SO16	549 ± 29	118 ± 5	272 ± 11	5 ± 0	117 ± 9	549.3	241.7	0.30	1.19	1.48
SO17	499 ± 20	210 ± 9	236 ± 4	9 ± 1	194 ± 30	585.7	260.3	0.32	1.28	1.58
SO18	500 ± 30	113 ± 5	251 ± 5	6 ± 0	133 ± 9	510.3	224.6	0.28	1.10	1.38
SO19	965 ± 57	127 ± 3	271 ± 11	6 ± 4	137 ± 83	589.2	262.8	0.32	1.29	1.59
SO20	898 ± 50	92 ± 4	176 ± 7	ND	ND	412.6	186.2	0.23	0.91	1.11
SO21	910 ± 52	153 ± 7	213 ± 8	ND	ND	527.8	237.4	0.29	1.16	1.43
SO22	698 ± 49	107 ± 4	174 ± 4	ND	ND	410.7	184.2	0.23	0.90	1.11
SO23	809 ± 40	270 ± 20	213 ± 8	ND	ND	637.1	287.2	0.35	1.41	1.72
SO24	728 ± 39	150 ± 12	87 ± 2	ND	143 ± 21	330.5	152.2	0.19	0.75	0.89
SO25	868 ± 49	53 ± 2	59 ± 2	ND	49 ± 8	203.7	96.1	0.12	0.47	0.55
Min	136	53	58.8	ND	ND	203.7	96.1	0.12	0.47	0.55
Max	1269	288	272	9	215	715.9	321.2	0.39	1.58	1.93
SD	272	58	67	3	62	135.6	59.4	0.07	0.29	0.37
Arth.Mean	671	124.9	157	6	99	416.9	188.2	0.23	0.92	1.13
World.Mean						370	60	0.07	0.46	

*ND* not detected

### Instrumentation and calibration process

#### Instrumentation

The activity concentrations of ^226^Ra, ^232^Th, ^40^K, ^235^U and ^238^U in soil samples were measured using high resolution gamma-ray spectrometric system. The system is consisted of a characterized Broad Energy (BE6530) germanium detector with active volume of 6500 mm^3^, relative efficiency of 60 % at 1.33 MeV ^60^Co line and a resolution of 2.2 keV at the same line. The detector is enclosed with a 10 cm thick cylindrical lead shield (Model 747 lead shield) to reduce the background radiation from various natural radiation sources and to isolate from other radiation sources used in nearby surrounding. The lead shielding is graded with an inner layer of 1 mm tin and 1.6 mm thick copper to reduce the contribution from Pb X-ray florescence and to prevent the effect of X-rays peaks in the background spectrum.

#### Calibration of the instrument and activity concentration

**Fig. 1:**
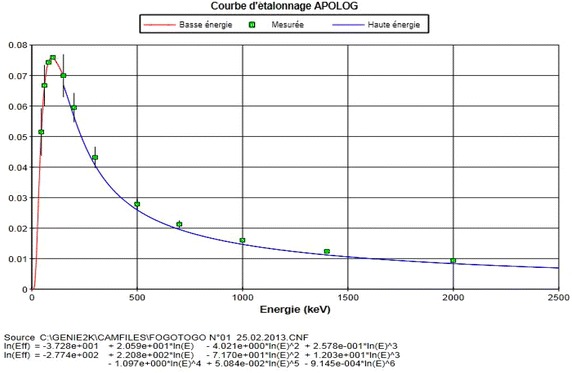
Efficiency calibration curve

The energy calibration of the system was done using point sources of ^109^Cd, ^57^Co, ^137^Cs and ^54^Mn. The counting time was adjusted to record at least higher counts for each full-energy peak in order to minimize the statistical counting error. The absolute efficiency of the system was performed using ISOCS/LabSOCS mathematical calibration software in build with Monte Carlo. Efficiency calibration file was generated using Geometry Composer Tool in the software. In generating this, accounts were taken all parameters related to the measurement including dimensions of the counting geometries, physical and chemical compositions, and density of each sample as well as the distance source-to-detector end-cap including attenuation, absorption and cascade summing correction. In validating the accuracy of the mathematical efficiency calibration software, some test were conducted comparing the ISOCS/LabSOCS generated efficiency results with the empirical peak efficiency for a ^60^Co point source positioned at a distance of 25 cm from the detector end-cap. The calculated results were in good agreement showing that differences between mathematical and empirical peak efficiencies are within 3–5 %. This validation of efficiency calibration files was done at the IAEA laboratory in Vienna through technical cooperation. To avoid error due to extrapolating the curve, the calibration curve was plotted in dual mode with cross-over energy at 165.85 keV (^139^Ce). A fourth order polynomial equation with the best fit for the lower and higher energy curve were obtained (Fig. 1) for the energy less than 165.85 keV (see the read part of the curve) and for the energy greater than 165.85 keV (the blue part of the energy curve).

To measure activity concentration of nuclides in samples, each sample was counted for a time ranging between 24 and 48 h for effective peak area statistics of above 0.1 %. Spectra were analyzed off-line using Genie 2000 Version V.3.2.1, including peak search nuclide identification activity and uncertainty calculation and Minimum Detection Limit (MDA) of gamma ray system at 95 % confidence level calculation modules software based on the equation taken from Aoun et al. (2015). This was as followed:

1 $$MDA(Bq/kg) = \frac{4.66 \times \sqrt B }{{\varepsilon \times P_{\gamma } \times T \times M}}$$

where B is the background counts, ε is the absolute efficiency of the detector, Pγ is the gamma emission probability, T is the counting time, M is the mass of the sample assuming an average mass of all samples equal to 0.12 kg.

The background was frequently measured under the same conditions applied on the samples (48 h) and used to correct the net count rate of gamma rays of measured isotopes and to evaluate the minimum detection activity of isotope. These were 26.05 Bq kg^−1^ for ^40^K, 7.55 Bq kg^−1^ for ^226^Ra and 10.25 Bq kg^−1^ for ^232^Th. The activity concentrations were averaged from gamma-ray photopeaks at several energies assuming the secular equilibrium between ^226^Ra and ^222^Rn daughters ^214^Pb and ^214^Bi, and ^232^Th and ^228^Ra. ^235^U activity concentration was determined using its direct gamma-rays photopeak 185.7 keV, interference correction due to the presence of 186.2 keV energy peak of ^226^Ra was taken into account and subtracted accordingly. To determine the activity concentrations of ^238^U in the samples, the gamma-ray photopeaks of ^234^Th and ^234m^Pa were used.

### Radiological risk study

As a result of non-uniform distribution of ^226^Ra, ^232^Th and ^40^K in soil, uniformity with respect to exposure to radiation has been defined in terms of radium equivalent activity (Ra_eq_) in Bq kg^−1^ to compare the specific activity of materials containing different amounts of ^226^Ra, ^232^Th and ^40^K. This was evaluated using Eq. (2) taken from Uosif et al. (2015); Gonzalez-Fernandez et al. (2012).

2 $$Ra_{eq} = A_{{{}^{{226_{Ra} }}}} + 1.43 \times A_{{232_{Th} }} + 0.077 \times A_{{40_{K} }}$$

where *A*_*Ra*_, *A*_*Th*_ and *A*_*K*_ are the activity concentrations of ^226^Ra, ^232^Th and ^40^K in soil samples.

The absorbed gamma dose rate outdoors at a height of 1 m above the ground surface can be evaluated from the activity concentrations of the ^226^Ra, ^232^Th and ^40^K measured in soil samples using the formula given in UNSCEAR (2000).

3 $$D\left( {{\text{nGy}}\,{\text{h}}^{ - 1} } \right) = 0.462A_{Ra} + 0.604A_{Th} + 0.0417A_{K}$$

where *D* is the absorbed dose rate in nGy h^−1^, *A*_*Ra*_*, A*_*Th*_ and *A*_*K*_ are the activity concentration of ^226^Ra (^238^U), ^232^Th and ^40^K, respectively. The dose coefficients in units of nGy h^−1^ per Bq kg^−1^ were taken from the UNSCEAR (2000).

From the fact that population in the area used concrete bricks as building materials, two external exposure scenarios were considered; outdoor (living within and near the study area) and indoor (living in house around built with concrete bricks made of soil) exposure. The annual outdoor and indoor effective doses in units of mSv per year were then computed using conversion coefficient factors from absorbed dose in air to effective dose taken from UNSCEAR (2000).

For indoor:

4 $$Annual \, effective \, dose\,\left( {{\text{mSv}}\,{\text{y}}^{ - 1} } \right) = {\text{D}}\left( {{\text{nGy}}\,{\text{h}}^{ - 1} } \right) \times 8760\;{\text{h}} \times 0.8 \times 0.7\,{\text{Sv}}.{\text{Gy}}^{ - 1} \times 10^{ - 6}$$

For outdoor:

5 $$Annual\,effective\,dose\,\left( {{\text{mSv}}\,{\text{y}}^{ - 1} } \right) = {\text{D}}\left( {{\text{nGy}}\,{\text{h}}^{ - 1} } \right) \times 8760\;{\text{h}} \times 0.2 \times 0.7 \times {\text{Sv}}\;{\text{Gy}}^{ - 1} \times 10^{ - 6}$$

The values of those parameters used in the UNSCEAR (2000) are 0.7 Sv Gy^−1^ for the conversion coefficient from absorbed dose in air to effective dose received by adults and 0.8 for the indoor occupancy factor and 0.2 for the outdoor occupancy factor.

Another criterion used to estimate the level of gamma ray radiation associated with natural radionuclides in specific construction materials is defined by the terms External hazard index (Hex) as shown in Eq. (6) obtained from UNSCEAR (2000).

6 $$H_{ex} = \frac{{A_{Ra} }}{370} + \frac{{A_{Th} }}{259} + \frac{{A_{K} }}{4810} \le 1$$

where *A*_*Ra*_, *A*_*Th*_ and *A*_*K*_ are the activity concentrations of ^226^Ra, ^232^Th and ^40^K in soil samples.

## Results and discussion

### Radiometric analysis

**Fig. 2:**
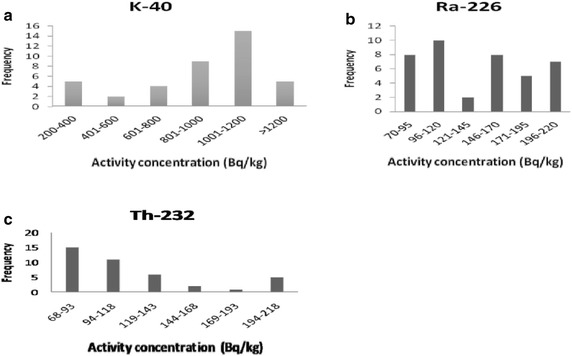
Frequency distributions of ^40^K (**a**), ^226^Ra (**b**) and ^232^Th (**c**) in soil samples

Activity concentrations of ^40^K, ^226^Ra, ^232^Th, ^235^U and ^238^U measured in the soil samples together with the statistical error are given in Table 1. Activity concentrations of ^40^K, ^226^Ra and ^232^Th ranged from 136 ± 2 to 1269 ± 128 Bq kg^−1^ with a mean of 671 ± 272 Bq kg^−1^, 53 ± 2 to 288 ± 22 Bq kg^−1^ with an average of 125 ± 58 Bq kg^−1^, 59 ± 2 to 272 ± 11 Bq kg^−1^ with a an average of 157 ± 67 Bq kg^−1^, respectively. The activity concentration of ^238^U ranged from ND (not detected) to 215 ± 40 Bq kg^−1^ with an average of 99 ± 62 Bq kg^−1^. It can be seen in Table 1 that ^235^U was detected in twelve (12) samples. The low activity concentration of ^235^U compared of that of ^238^U in the samples is attributed to the abundance of both isotopes in the entire earth i.e. ^235^U (0.75 %) and ^238^U (99.8 %). Activity concentration of ^235^U is low with an average value of 5.8 ± 2.7 Bq kg^−1^. The low activity concentration of ^235^U compared to that of ^238^U in the samples is attributed to the abundance of both isotopes in the entire earth i.e. ^235^U (0.75 %) and ^238^U (99.28 %). The observed ratio between ^235^U and ^238^U in the samples was 0.045. The activity concentration of ^40^Kwas observed to be comparatively higher than that of both ^226^Ra and ^232^Th in all the soil sampling locations of the studied area. The radioactivity of primordial radionuclides of soil generally depends on the local geological media which they are derived, the mineral deposition and the transfer processes. In cores of such geological rocks and soil formation, physical disaggregation and geochemical interaction influences the distribution of primordial radionuclides and their decay products and ^40^K. The rocks types fund in this site are essentially the basement rocks (orthogneiss, gneiss, migmatites, alkali feldspar, goethite, hematite, kaolinite and amphibolites) and various volcanic rocks (basalts, granites, trachytes, phonolites, tuffs, breccia and ignimbrites). Some of this basement and volcanic rocks such as gneiss, granites, basalts and orthogneiss are known to have more of primordial radionuclides than other. Figure 2 shows the frequency distribution of ^40^K, ^226^Ra and ^232^Th activity concentrations in the soil samples. The ranges of 96–120 and 68–93 Bq kg^−1^ measured for ^226^Ra and ^232^Th, respectively (25 % for ^226^Ra and 37.5 % for ^232^Th) include most samples. The activity concentrations of ^40^K measured in 37.5 % of the total soil samples are between 1000 and 1200 Bq kg^−1^.

The specific radioactivity of ^226^Ra, ^232^Th and ^40^K determined for soil samples were compared with the available data from other countries and Earth’s crust as seen in Table 3. As seen in Table 3, activity concentration of terrestrial radionuclides found in soils varies from country to country. However, the values reported in Table 3 are not representative for the countries mentioned but only for the region in which the samples were collected. The obtained values of activity in this study are relatively high except that of ^226^Ra published by Carvalho et al. (2007) and by Winkelmann et al. (2001) and those of ^40^K published by Uosif (2011) in Upper Egypt. This variation in radioactivity could result from the type of mineral ore deposit and the geotechnical characteristic of the area.

Comparing the average of ^40^K, ^226^Ra and ^232^Th in the soil samples with the current worldwide mean in normal continental soil (Table 2), the obtained means are over 73.56 % for ^226^Ra, 71.39 % for ^232^Th and 38.59 % for ^40^K than the average worldwide. This can be justified because the investigated soil samples are from the bauxite core deposit which from the literature usually contains natural high radioactivity compare to the normal zone (IAEA 1996). In addition to that, mineralogical study of the area also reveals the presence of rocks and minerals associated with natural radioactivity. The high activity of ^40^K is because the rock ore of the mine is associated feldspar which belongs to a group of hard crystalline minerals that consist of aluminium silicates of potassium, sodium, calcium. Even though the average values of primordial radionuclides in the present study are higher than the worldwide values reported by UNSCEAR (2000), the specific activity of ^40^K, ^226^Ra and ^232^Th are still far below the exemption levels recommended in the basics safety standards (IAEA 1996).

**Table 2:** Comparison of the activity concentration of terrestrial radionuclide with other published values

Location	Activity concentration (Bq kg^−1^)			References
	^226^Ra	^232^Th	^40^K	
South India (Gudalore)	17–62	19–272	78–596	Selvasekarapandian et al. (1999)
NorthenIndia (Upper Siwaliks)	28.3–81.0	61.2–140.3	363.4–1002	Singh et al. (2005)
Upper Egypt	31–40	52–61	3149–3210	Uosif (2011)
China	1–360	2–690	9–1800	UNSCEAR (2000)
USA	4–130	4–140	100–700	UNSCEAR (2000)
Camroon (volcanic area)	14.00	30	103	M. Ngachin et al. (2008)
Syria	19.00	24	336	Al-Marsi et al. (2006)
Turkey	28.60	33	448.5	Turhan et al. 2012
Portugal (Uranium mining)	200.00	91	–	Carvalho et al. (2007)
Eastern Germany (Ronneburg)	370.00	45	620	Winkelmann et al. (2001)
Brazil (Rio Grande do Norte)	29.20	47.8	704	Malanca (1996)
Cameroon (Mini-Matap)	53–288 (125)	59–272 (157)	136–1269 (671)	Present study
World average	16–116 (33)	7–50 (45)	100–700 (420)	UNSCEAR (2008)

### Radiological risk study

The maximum value of Ra_eq_, in building materials as well as geological material must be less than 370 Bqkg^−1^ for the material to be considered safe for use ICRP (1996). From Table 2, radium equivalent activity (Ra_eq_) varied from 203.7 to 715.9 Bq kg^−1^ with a mean of 416.9 ± 135.6 Bq kg^−1^ which is over 11.25 % than the worldwide average. This implied that using of soils in the area as building material might present significant radiological health risk. No uniform trend in the variation of terrestrial radioactivity has been found from the studied area.

Calculated values of the absorbed dose rate (AD), outdoor Annual Effective Dose, indoor annual effective dose and external hazard index (H_ex_) of soil samples are presented in Table 1. The total absorbed dose rate in air varied from 96.1 to 321.2 nGy h^−1^ with an average of 188.2 ± 59.4 nGy h^−1^. The obtained mean value is over 3.14 times than the reported population-weighted mean value of 60 nGy h^−1^ for regular area given by UNSCEAR (2000). This difference in absorbed dose rate with the reported values of UNSCEAR could be attributed to differences in geology formation and geochemical structure of the sampling sites. The contribution of each primordial radionuclide to the average gamma absorbed is given in Table 3. It can be seen that ^232^Th contributes more to the absorbed dose rate in air.

**Table 3:** Natural radionuclides in soil and the corresponding absorbed dose rate in air 1 m above ground

Nuclide	World average (UNSCEAR 2008)		Fongo-Tongo (study area)		
	Concentration (Bq kg^−1^)	Dose rate (nGy h^−1^)	Concentration (Bq kg^−1^)	Dose rate (nGy h^−1^)	% contribution
^226^Ra	33	25	124.85	28.58	15.40
^232^Th	45	45	157.27	53.31	28.33
^40^K	420	30	670.92	104.11	55.32

The values of the outdoor and indoor annual effective dose in soil samples varied from 0.12 to 0.39 mSv year^−1^ with a mean of 0.23 ± 0.07 mSv year^−1^ and from 0.47 to 1.58 mSv year^−1^ with an average of 0.92 ± 0.29 mSv year^−1^, respectively. The worldwide average annual effective dose rate is approximately 0.5 mSv year^−1^ and the results for individual countries being generally within the 0.3 to 0.6 mSv year^−1^ for indoors. The observed values for outdoor in this study are within the range while that of indoor are relatively high. The recommended average annual for an individual member of the public by the International Commission on Radiological Protection is 1 mSv year^−1^. In the present study, most of the observed total annual effective doses are higher that the recommended value of ICRP (1991) with the average approximately equal to the safe limit. As seen in Table 1, the obtained value of the external hazard index in the investigated soil samples ranged from 0.55 to 1.93 with a mean of 1.13 ± 0.37 which is slightly higher than the published value by UNSCEAR (2000) for the protection of the public.

## Conclusion

In the present study, activity concentration of ^226^Ra, ^232^Th, ^40^K, ^235^U and ^238^U in the surface soil samples from bauxite ore deposit site in Dschang was determined by using high resolution gamma spectrometry based Broad Energy Germanium (BEGe6350) detector. The observed activity concentrations means of radionuclides are over 73.56 % for ^226^Ra, 71.39 % for ^232^Th and 38.59 % for ^40^K than the reported worldwide means by UNSCEAR. However lower than the recommended safe limit of mineral bauxite ore deposit zone reported in the IAEA International basic safety standards (BSS) for protection against ionising radiation and for the safety of radiation sources. To assess possible radiological hazard, absorbed gamma dose rate in air (AD), the corresponding outdoor and indoor annual effective dose and the external hazard index (H_ex_) have been estimated. The calculated means of these radiological risk parameters are marginally higher than the worldwide range. For radiological safety impact parameters such as radium equivalent activity and external hazard index, using of soil in the area as building materiel might increase radiation risk to the population and data obtained in this study could be used as a baseline data for preparing radiological map of the study area.
